# Initial study on an expert system for spine diseases screening using inertial measurement unit

**DOI:** 10.1038/s41598-023-36798-7

**Published:** 2023-06-27

**Authors:** Mariusz Pelc, Radana Vilimkova Kahankova, Monika Blaszczyszyn, Dariusz Mikolajewski, Mariusz Konieczny, Volodymir Khoma, Gregor Bara, Jaroslaw Zygarlicki, Radek Martinek, Munish K. Gupta, Edward Jacek Gorzelanczyk, Mateusz Pawłowski, Bogdan Czapiga, Malgorzata Zygarlicka, Aleksandra Kawala-Sterniuk

**Affiliations:** 1 Faculty of Electrical Engineering, Automatic Control and Informatics, Opole University of Technology, 45-758 Opole, Poland; 2 School of Computing and Mathematical Sciences, University of Greenwich, London, SE10 9LS UK; 3 Faculty of Electrical Engineering and Computer Science, VSB-Technical University of Ostrava, Ostrava–Poruba, Czech Republic; 4 Faculty of Physical Education and Physiotherapy, Opole University of Technology, 45-758 Opole, Poland; 5 Faculty of Computer Science, Kazimierz Wielki University, 85-064 Bydgoszcz, Poland; 6 Lviv Polytechnic National University, Institute of Computer Technologies, Automation and Metrology, Lviv, Ukraine; 7 Department of Neurosurgery, University Hospital Bonn, Bonn, Germany; 8 Faculty of Mechanical Engineering, Opole University of Technology, 45-271 Opole, Poland; 9 Department of Mechanical Engineering, Graphic Era University, Dehradun, India; 10 Faculty of Philosophy, Kazimierz Wielki University, Bydgoszcz, 85-092 Poland; 11 Faculty of Mathematics and Computer Science, Adam Mickiewicz University in Poznan, Poznan, 61-614 Poland; 12 Department of Theoretical Basis of Biomedical Sciences and Medical Informatics, Nicolaus Copernicus University, Collegium Medicum, 85-067 Bydgoszcz Poland; 13 The Society for the Substitution Treatment of Addiction “Medically Assisted Recovery”, 85-791 Bydgoszcz, Poland; 14 Psychiatric Department of Children and Adolescents Psychiatric Center in Warta, 98-290 Warta, Poland; 15 Faculty of Health Sciences, Wroclaw Medical University, Wrocław, Poland; 16 Department of Neurosurgery, “Vital Medic” Hospital, Kluczbork, Poland; 17 Department of Neurosurgery, 4th Military Hospital in Wrocław, Wrocław, Poland

**Keywords:** Biomedical engineering, Preclinical research

## Abstract

In recent times, widely understood spine diseases have advanced to one of the most urgetn problems where quick diagnosis and treatment are needed. To diagnose its specifics (e.g. to decide whether this is a scoliosis or sagittal imbalance) and assess its extend, various kind of imaging diagnostic methods (such as X-Ray, CT, MRI scan or ST) are used. However, despite their common use, some may be regarded as (to a level) invasive methods and there are cases where there are contraindications to using them. Besides, which is even more of a problem, these are very expensive methods and whilst their use for pure diagnostic purposes is absolutely valid, then due to their cost, they cannot rather be considered as tools which would be equally valid for bad posture screening programs purposes. This paper provides an initial evaluation of the alternative approach to the spine diseases diagnostic/screening using inertial measurement unit and we propose policy-based computing as the core for the inference systems. Although the methodology presented herein is potentially applicable to a variety of spine diseases, in the nearest future we will focus specifically on sagittal imbalance detection.

## Introduction

Nowadays, due to civilisation changes in people lives and especially lack of physical activity or overweight, many people suffer various kinds of degenerative spine diseases^1–7^. Before a proper therapy can be applied, there is utmost importance of proper diagnosis of which specific disease type a patient suffers from^8–10^. For this reason, there is a huge demand for accessing the necessary medical procedures which would allow unequivocal identification of the disease type and provide adequate therapy^6,8,10–13^. Hence diagnosis based on X-Ray or CT, surface topography or MRI scans is needed^11,14,15^. However, since these are extremely costly diagnostics and in some cases accessing them requires long waiting list due to limited capacity and increased demand waiting of patients (X-Ray or CT or MRI scan is also commonly used in diagnostic of many other than spine-bone related diseases), many patients cannot access timely and adequate treatment^6,12,13^. This results in various negative effects in, as the bad posture or degeneration of the spine-bone related diseases have tendency to deepen and most of all – they are usually associated with overwhelming pain^13,16^. Another method, which is currently less popular, but also non-invasive is surface topography (ST). This method allows vertebral orientation recording in 3D^15,17^. It is important to mention that the use of ST technology does not expose the patient to harmful radiation, unlike X-Ray^15,17,18^. In various spine diseases – repeated exposure to X-Ray may be harmful for patients^15,17–20^.

Its advantage is that it can be carried out in both static and dynamic situations, where patient moves for the examination purposes^17,18,20^. This allows various kinematic patterns to be assessed in three dimensions^17^. It can also be considered a valuable screening tool as it is non-invasive, not harmful, so the examinations can be repeated numerous times^15,17,19^.

It is possible to distinguish between static and dynamic assessment of spinal parameters for diagnostic purposes^20–23^. Diagnostic imaging methods can be divided into dynamic and static assessment, where the dynamic one involves comparing patients’ various positions^21^. The surface topography allows dynamic examinations^17^. It is important to mention as stating imaging can only provide limited diagnostic information as it delivers temporal snapshots only^22^. For the purpose of spine shape, mobility and condition assessment, non-invasive measurements, which are not based on radiation, became very important, in particular dynamic measurement methods^20,23,24^.

Before the patient’s condition deteriorates to the point where only very expensive medical procedures (e.g. spine surgery, disc fusion, or disc implantation) are of any help, there is a lot of time for screening tests for early identification of any potential posture or spine-bone related problems. This way, significantly less expensive and minimal invasive and conservative treatment, such as physiotherapy, physical exercise, can be used^13,16,25^. The only problem is that, as mentioned before, these screening tests cannot be performed using all these expensive devices (like magnetic resonance) and tools for accessing which the patients with some more urgent health conditions are usually given priority^25^.

Balance is defined as the ability of the human body to maintain the centre of mass (COM) at the base of its support with minimal swaying^26–30^. The equilibrium cone refers to the stable area of upright standing^26–28,31–33^. The basic premise is that going beyond the individual cone challenges the mechanisms of equilibrium^26–28,33^ The most frequently observed changes include the spine, pelvis and lower limbs to compensate for the altered posture^26–28,31,33,34^.

The spine and other segments of the human body function within the cone of equilibrium, focusing on maintaining sagittal and coronal alignment with minimal energy expenditure^35^. This happens with a harmonious relationship involving cervical lordosis (CL), thoracic kyphosis (TK), lumbar lordosis (LL), and pelvic angles^26–28,35–38^. The main goal is to maintain the mechanical balance in the sagittal and coronal planes centered on the center of the mass of the skull, femoral heads and lower limbs^37,39–41^. Many authors report that sagittal balance, rather than coronal balance, is significantly correlated with quality of life^26–28,37,41–43^.

Therefore, more attention is paid to sagittal balance than to coronal balance in assessing spinal deformity, surgical planning, and surgery^26–28,44–46^. Overall, sagittal imbalance results in increased muscle exertion and energy expenditure, causing pain, fatigue and disability^26–28,44,46^. Sagittal spinal imbalance as a key factor in the pathogenesis of myelopathy is supported by several reports^26–28,44,47–49^. Many studies have described the normative values of the pelvic spine alignment parameters in various populations of different ages and pathological conditions^26,27,27,28,50^. These studies have well established the correlation of pelvic parameters, TK, sagittal imbalance and their impact on quality of life and treatment outcomes. Many studies have reported that pelvic incidence (PI), a constant morphological parameter in each individual, has a significant effect on the sagittal alignment of the lumbar or thoracic spine, such as LL and CT^51–54^. The correlation between the parameters of the cervical and thoracic spine is weaker than between the parameters of the lumbar spine and the pelvis^26–28,53,55,56^.

This is where the approach proposed in this paper may come in help. The solution is based on using Inertial Measurement Units (IMUs) as a low-cost of-the-shelf device which could be used as an alternative method to the state-of-the-art diagnostic methods, which are expensive and not ad-hoc accessible. More and more IMU-based systems for spine condition assessment are being proposed^57–59^; in particular for sitting posture detection^57^. Mostly due to the rapid development of Internet of Things and/or edge computing, which has led to the development of inexpensive systems, such as inter alia those IMU-based^57^, which are becoming more and more popular not only because of their price but also for their ability for movement qualitative examination^58–60^. Despite some flaws in data quality, they have potential for becoming a very useful tool for clinicians as they can be applied for inter alia dynamic assessments^58,60^.

Also, females affected with scoliosis are in a group with higher risk of cancer due to the necessity of repeated X-Ray measurements^61–63^, which may be carcinogenic due to radioactivity and the fact that patients are exposed to it multiple times^64^. The use of an IMU-based assessment system may reduce this risk, as IMUs are fully non-invasive and not radioactive.

As mentioned above – the main aim of this paper was to bring closer the use of IMU sensors in the diagnosis of the spine as an alternative to more expensive, invasive, harmful radiation methods for spine diseases screening.

This work comprises 7 following sections: Introduction explaining rationale of the study, Related work providing context for the whole research, Materials and Methods providing information about the hardware used and the way we approached the problem, Results presenting acquired data, Discussion where we critically discuss the results and, Conclusion providing summary of the research and the key findings and finally References section listing all the sources relevant for our study.

## Related work

One can find a very wide selection of publications when it comes to diagnostic of various kinds of spine-related issues using typical clinical devices (X-Ray, MRI, Tomography, surface topographyetc.)^23,24,65,66^. These devices are allowing the clinicians to obtain a very high quality image/scan of the disease-affected area and automate the diagnostic process by involving image processing methods. By proportion, vast majority of the papers use machine learning/deep learning methods to diagnose and/or monitor spine-related diseases including some higher level functional problems e.g. related to spine deformations as well as some very specific ones, e.g. related to disc herniation or spinal myelopathy, e.g.^67–71^. Many publications, beginning from early 2000s, were focusing on using various classification methods to classify some specific spine diseases^72–74^. The common numerator of these all publications is that they are using imaging provided by traditional clinical devices as a source of information about the spine condition.

Available publications related to spine diseases diagnostic reveal that performing them other than by using a qualified clinical equipment (e.g. X-Ray, MRI) is rather untypical (the least to say), especially when compared by proportion with the huge variety of studies based on spine imaging, and seems in its infancy. But it looks that e.g. IMU-based diagnostics has gained some momentum, and for a good reason: on one hand, using the clinical equipment generates sometimes huge cost but on the other hand, as of today, this is the only equipment used and all diagnostic method are based on the results it provides. Actually, this equipment is the foundation on which the whole teaching in medical sciences.

However, gradually there are more and more attempts to provide alternative approaches which will be less costly, better applicable especially to *en masse*/screening purposes but they will still guarantee enough accuracy to become a valuable and trustworthy diagnostic tool.

One of the attempts where IMU was used as a device for human motion analysis was described in^59^. In these publications the Authors acquired signals from a set of IMUs embedded into a vest and then worn by a patient. These signals were analysed using statistical methods to identify *axial spondyloarthritis*. Very similar method focusing on monitoring lower back area was also described in^75^.

Another example of using IMU for detection of spine-related diseases (specifically, axial spondyloarthritis) can be found in^76^. The Authors, similarly to our approach, decided to place electrodes along the spine and then record signals from a number of IMUS. The signal was then analysed using Bland-Altman analysis along with statistical analysis in order to assess the level of agreement between measurements. What makes this approach different from our one is that the Authors have come up with a fixed and not easily adjustable method which does not leave much space for adjustments, should a need arise. Most importantly, this approach does not qualify as an expert system due to the fact that there is no mechanism allowing expressing/transferring/updating expert knowledge so that it would affect changes in the whole system behaviour.

In^77^ the Authors targeted evaluation of *ankylosing spondylitis* using a wearable system with multiple (16) magnetic and IMU units. Signals acquired from the sensor fusion were used to determine maximum *RoM* (Range of Movement) of lateral and cervical flexion and based on this evaluate overall spine mobility. This whole system provided very high accuracy (around $90 \%$) and as such can qualify as a clinical diagnostic tool. However, despite its accuracy it focused only on a narrowed diagnostic spectrum and being relatively “fixed” structurally, it does not offer an easy way of adjustment or targeting a different diagnostic spectrum as this would require very substantial changes in the system design and as such, it does not provide an interface for doing this via a formalised (e.g. in a form of a policy) expert knowledge transfer.

An interesting study is included in the^78^ where Authors use a set of small unintrusive sensors to detect rheumatic and musculoskeletal diseases. Authors focus specifically on the lower back area and use 3 horizontally placed sensors to acquire relevant measurements. The interesting angle of this study is that in this 3-unit system only 2 sensors are effectively used to record signals: one IMU sensor is used to measure the acceleration(s) and body position while the other sensor is destined to measure muscle activity. The third unit is used solely for signal processing. So, this solution on one hand provides less acceleration/body position related information but on the other hand, it adds to this a completely different category of information which could allow a better understanding not only skeletal-related issues but also those appearing at the muscular levels.

In^60^ the Authors evaluate IMU as a potentially useful tool which clinicians and researchers might use for spine-related disease diagnosis. They use IMU to acquire signals allowing them to assess local dynamic stability and variability through statistical analysis. This is just another approach which is using a statistical apparatus which is tuned for a purpose but does not qualify as an expert system because there is no easily available interface for altering the system behaviour through transfer of updated knowledge.

## Materials and methods

Inertial Measurement Unit (IMU) is a device which is usually equipped with a number of measurement components:

- Gyroscope is a device that is used for measuring (or in some applications – maintaining) orientation and angular velocity;
- Accelerometer is a device that is typically used to measure motion by converting physical movement into an electrical signal; this signal can be measured recorded or analysed for various purposes;
- In some cases IMU can be additionally equipped with:
  - Magnetometer which is a device used to measure the strength and direction of the magnetic field in the vicinity of the instrument (so as such magnetometer allows to determine or maintain orientation along magnetic field axes);
  - Temperature sensor allowing to measure an ambient (or patient) temperature;
  - Pulse oximeter allowing to measure oxygen desaturation of the blood and pulse rate and hence allowing to determine factors like e.g. relaxation or stress levels, detect hypoxia, etc.

Now, as far as IMUs are concerned, there is quite a selection of these devices yet not all of them are equally good for the fore-mentioned purpose. This may be for many different reasons including signal quality, build quality, dimensions, availability of software which could be used for signal acquisition purposes (e.g. some of the IMUs are coming together with such software whereas some other IMUs require writing such a software entirely from scratch). Also, there are some other factors which may exclude some of the available products from use, such as e.g. availability of after-sale support, price, compatibility with typical embedded systems (e.g. Arduino or Raspberry PI), etc.

**Figure 1:**
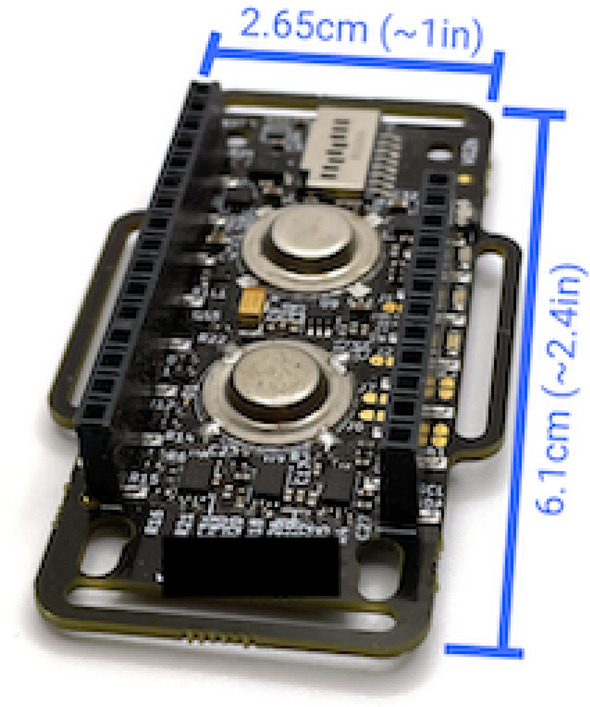
IMU unit^79^.

After thorough investigation of available IMUs we have decided to use a higher quality IMU which for which its manufacturer^79^ provides a very good variety of software (see Fig. 1).

This unit (see: Fig. 1) allows measuring $16+$ biosignals so in case a need would arise, not only the traditional IMU sensors can be used (accelerometer, gyroscope and magnetometer) but may others which can potentially improve the diagnostics spectrum.

Full list of the IMU Parameters is listed below:

- Sensor Sampling Rate:
  - 25 Hz – 9-axis IMU (accelerometer, gyroscope, Magnetometer),
  - 25 Hz PPG (photoplethysmography),
  - 15 Hz EDA/GSR (electrodermal activity/galvanic skin response),
  - 7.5 Hz temperature.
- Size:
  - 2.4 in (6.10 cm) length,
  - 1.0 in (2.65 cm) width,
  - 3.4 in (2.00 cm) height.
- Platforms:
  - Windows,
  - Mac,
  - Linux,
  - stretch goal: Android app.
- Battery life:
  - $2.5-4$ hours live-streaming,
  - $6-9$ hours data-logging,
  - stretch goal: power optimisation.
- User-owned data:
  - $100 \%$ raw data + derivative metrics.
- Wireless protocols:
  - WiFi,
  - stretch goal: add Bluetooth capability,
  - Also possible from Adafruit: LoRa, 4G.
- Time-sync with other devices:
  - typically $<10$ ms (network-dependent).
- Open source:
  - FW, SW, HW (firmware, software, hardware) schematics,
  - Arduino and Adafruit Feather Ecosystem compatible.

Additionally, in order to get the best out of the IMU device, its manufacturer also provides the following software components:

- Oscilloscope – software which allows on-line visualisation of all the signals sensed by the device;
- DataParser – software which allows off-line processing of previously recorded data;
- Biometric Lib – a library of examples and/or functions which allows even more fine-grained processing of the IMU data in Python, Matlab, etc.;
- *dataviewer.py* – Python program which is used to display the signals; although this software is provided by the IMU manufacturer, the quality (readability) of the displayed information leaves a bit to be desired so it is worth to consider using an alternative software to display the results.

**Figure 2:**
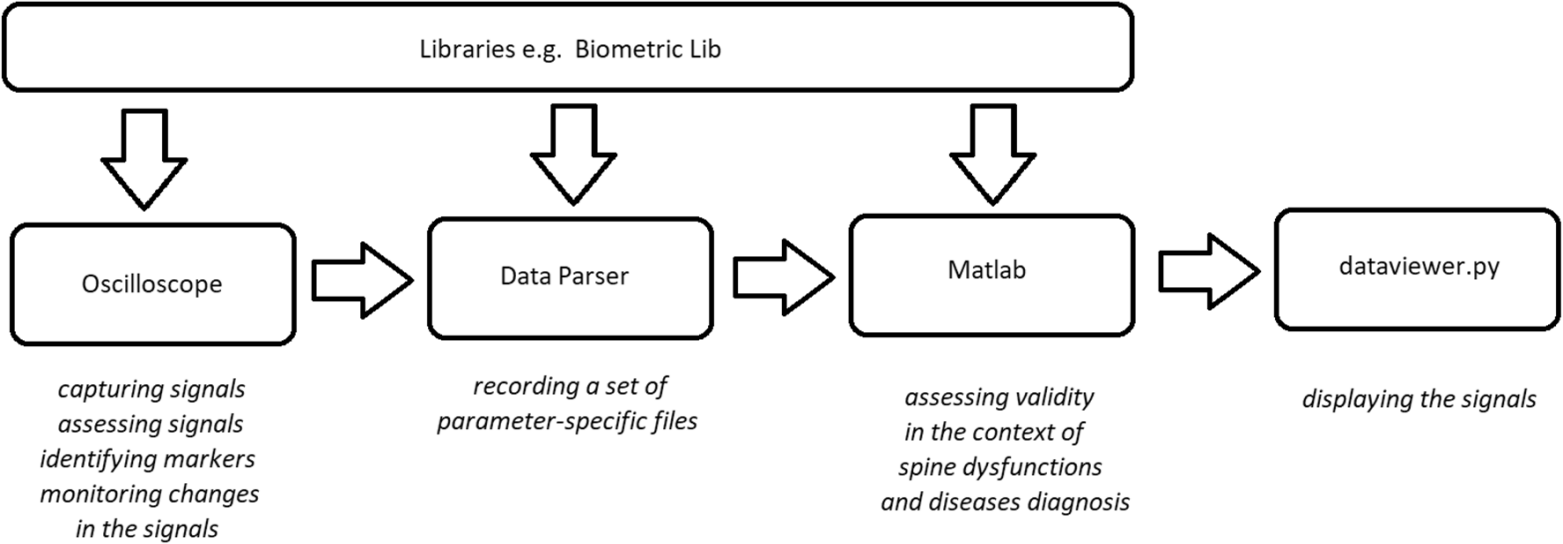
Summary of the the device software components functional interaction.

Having all the software available in this preliminary study we will mostly focus on using only the *Oscilloscope* tool to initially assess the signals and identify some markers which would be useful for diagnostic purposes and then we will perform a set of simple exercises thoroughly monitoring changes in the signals. Since the *Oscilloscope* tool allows also capturing the sensed signal values into a file, then the *DataParser* tool will be used to break the recording file into a set of parameter-specific files which then will be used in Matlab to perform some initial computations to assess their validity in the context of spine dysfunctions/diseases diagnosis ( Fig. 2).

### Applied research methodology

The whole concept of using the IMU device as a low-cost/off-the-shelf alternative for the very expensive (and of limited availability) clinical diagnostic devices such as X-Ray Scanner, Computer Tomography or Magnetic Resonance in the context of spine degeneration screening tests would assume that spine diseases manifest themselves in a number of different ways. For example the segment(s) of spine which is affected by a disease typically becomes stiffened (this happens as the result of increased muscles tension in the disease-affected area) and impacts neighbour segments so that the are trying to take over and compensate the function(s) of the affected segment^80–82^. This means that in some cases they may work outside of their physiological movement range which leads to their gradual overloading and degradation (kind of domino effect). For obvious reasons, a person with a stiffened and painful spine will develop a completely different movement pattern compared to a healthy individual.

Based on this assumption and moving at the signal level it is safe to assume that a healthy individual (and thus a degeneration-free spine) would generate a very specific set of signals while performing various kinds of exercises / movements (e.g. sit-ups, forward bend, trying to look straight for a per, etc.). This set of signal values may then be used as a reference / pattern to which signals from a tested subject might be compared. Based on the deviation from the pattern values (in case any such are identified) and after some computations (e.g. pattern recognition) it will be possible to distinguish which of the tested individuals shows symptoms of a spine bone disease and possibly (after some further analysis) which specific disease this might be. Such an approach allows relatively high volume tests to be done relatively quickly and inexpensively, but also without necessarily engaging qualified medical professionals who at the same time could engage with some more demanding medical procedures.

**Figure 3:**
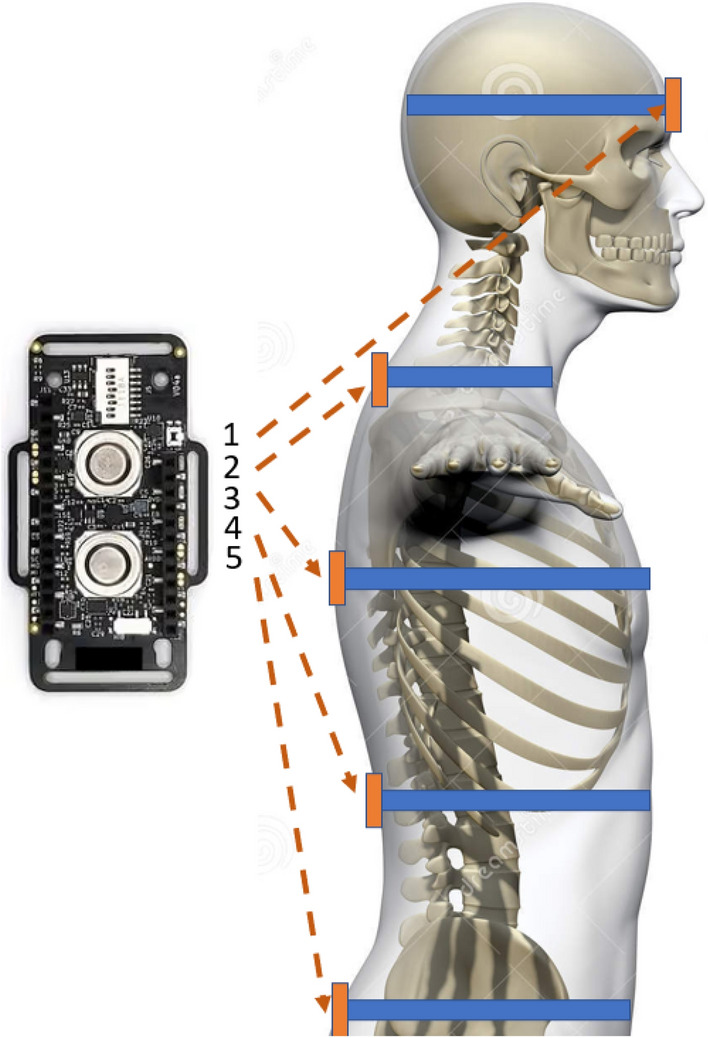
IMU placement^79^.

The key problem in using the IMU device for the screening and/or diagnostic purposes is to utilise information provided by the device so that it would properly serve the purpose. Since every single movement triggers a very complex reaction of the spine (various segments move in different plains, with different amplitudes, etc.) it becomes obvious that a single IMU device cannot provide enough information to serve as a foundation for building a reliable screening/diagnostic procedure. This is simply because only relative information (meaning how different spine segments move in relation to the other) would allow a better understanding of some functional deficiencies of the spine. Hence, other problems to address is deciding on how many IMU devices would be sufficient to provide enough information for the screening/diagnostic purposes or where specifically they should be placed. As there is no existing reference method that would help in this case, the only reasonable approach is to assume that the IMU devices should be somehow evenly distributed along the spine. And then (taking into account the *IMU* device dimensions) it seems that it is possible to fit in this way roughly something between 2-5 devices (depending on the patient’s height). Some possible places where the IMU units could be placed are shown in Fig. 3.

As far as IMU placement is concerned, limiting the number of IMU units used to only 2, we decided that one of them will be placed on the head and the other one in the upper chest area. While deciding about this specific placement wee looked at the spine as a specific kinetic chain and the IMU units were placed so that one would provide data originating from the end of the chain (IMU placed on head) and the other IMU was placed so that it was neither, too close nor too distant from the first one.

Another problem is to decide which specific method should be used for the purpose of the spine diseases (e.g. sagittal imbalance) detection/screening. On one hand the obvious solution might be e.g. using machine learning methods where an artificial neural network (ANN) could be employed to perform pattern recognition. However, using ANN has one big disadvantage: usually it decouples the ANN design/learning process, where usually an ANN design specialist expertise is needed, from the actual diagnostic process, which is usually done by a medical personnel. Typically, the medical personnel is lacking the expertise needed to make some even small adjustments to the inference system (e.g. repeat the whole learning process to make it population specific via repeating) and if such a need would arise the whole ANN design/learning process must be done from the beginning. What we propose instead is a very intuitive approach, where the medical personnel would know the core of the inference system and if it is needed, it can be easily adjusted without engaging the ANN expert. This is possible in case of policy-based systems because these systems use human-readable policy files reflecting expert’s (e.g. medicine doctor’s) knowledge to be used for diagnostic purposes. This approach on the contrary to the ANN-based approach does not require anyone else but the medical personnel (e.g. GP) to make required adjustments to the policy (via editing it using just a text editor) and as a result, improving the inference system accuracy.

It is important to mention, that in clinical practice most of the cases where spinal measurements are evaluated are based on (subjective) expert (clinical professionals) ratings^83–87^, despite the growing need for objective assessment methods development^85,86,88^. It means that for the current moment – modern, (Artificial Intelligence) AI-based methods do not replace human factors in the diagnosis process.

#### AGILE policies

AGILE Policy Definition Language (PDL)^89,90^ is a flexible and easy to grasp language which is used to express decision-making logic. It contains a number for functional elements (e.g. *Rules*, *ToleranceRangeChecks* or *UtilityFunctions* which provide a convenient construct to link a certain environment state (expressed by a number of *EnvironmentVariables* with an *Action* to be taken in case a (sub)set of relevant environment variables corresponds to e strictly defined system state. Since the term *system state* originates from the control theory area, it is important to understand how it is understood in the context of the diagnostic process and specifically, how it maps to symptoms indicating a specific disease. So, when a patient is being diagnosed, many symptoms are measurable whilst many others are not. E.g. we can easily detect a higher body temperature through a thermometer reading while measuring pain level is not that straightforward. But both, higher body temperature and pain in a given area may be symptoms of a disease. Now, state variables are meant by all these symptoms which are measurable directly or indirectly (meaning that they can be linked to some specific features extracted from e.g. signal measured using some measurement devices – in our case the IMU unit).

**Figure 4:**
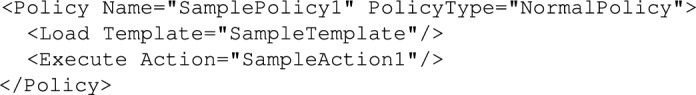
Example policy.

**Figure 5:**
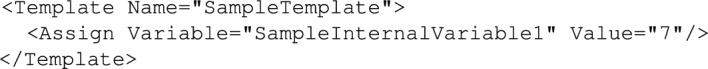
Example template.

**Figure 6:**
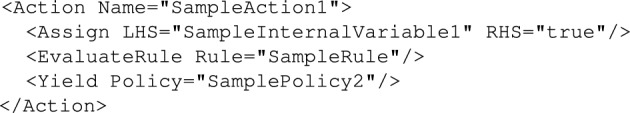
Example action.

**Figure 7:**
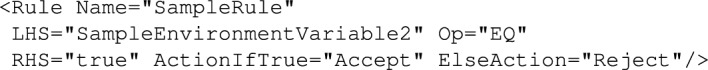
Example rule.

**Figure 8:**
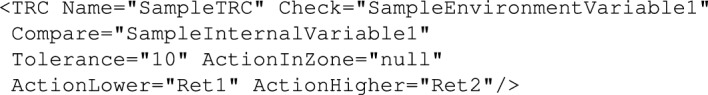
Example ToleranceRangeCheck.

**Figure 9:**
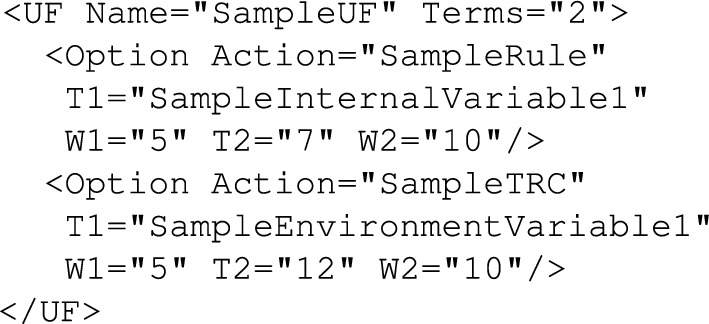
Example UtilityFunction.

AGILE PDL is based on XML schema and defines the following policy objects:

- *PolicySuite* contains all other policy objects.
- *Policy* defines decision making policy (one source policy file may contain zero or more policies). Policies can load *Templates* or execute *Actions*. Example *Policy* is shown in Fig. 4.
- *Template* is used to configure policies (for example, to assign some values to *InternalVariabies*). Example *Template* is shown in Fig. 5.
- *Action* gathers policy decision-making logic (it evaluates *Rules*, *TRCs*, *UFs*, it can also assign local variables or yield *Policies*) and finally returns a policy decision. Example *Action* is shown in Fig. 6.
- *Rule* is used to compare variables (*ExternalVariables* or *LocalVariables*), values, etc. Depending on the comparison result an appropriate *Action* is executed. Example structure of *Rule* is presented in Fig. 7.
- *ToleranceRangeCheck* (TRC) is an implementation of deadzone which is especially useful in case of tracking of a dynamic goal; the deadzone is specified as one of the TRC parameters. Depending on the value of the tracked parameter an appropriate action is executed. Example structure of *TRC* is presented in Fig. 8.
- *UtilityFunction* (UF) is used to indicate goal-attainment or want-satisfaction. Depending on the utility level an appropriate *Action* is taken. Example *UtilityFunction* is shown in Fig. 9.

**Figure 10:**
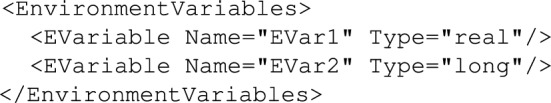
Example declaration of *ExternalVariables*.

Policies allow processing information about the system using *EnvironmentVariables* (see Fig. 10).

**Figure 11:**
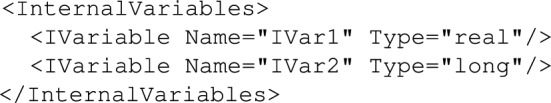
Example declaration of *InternalVariables*.

Some reference values (needed to compare system state variables values to some threshold values) or current information about the policy state (e.g. users choices or any information the policy reaches during its evaluation) can be stored using *InternalVariables* (see Fig. 11).

**Figure 12:**
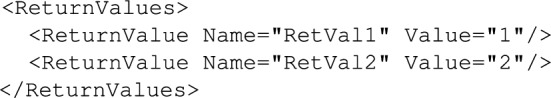
Example Declaration of *ReturnValues*.

Policies return decisions in through *ReturnValues* objects. Their example definition is provided in Fig. 12.

Policy decision can be read through a designated API function (*EvaluatePolicySUite*) provided in the *AGILE*_*Lite* library^90^. However, this (and other) relevant functions are part of a program running policies and the program can be provided for use by the medical personnel (this program has to basically record the signals, extract the relevant features and then pass them to the policy for evaluation purposes), so that the medical personnel could focus solely on the policies design/adjustments. Such a great flexibility (decoupling the core software design and policy design processes) is what makes policy-based systems so powerful compared to the other technologies used in expert systems because once there signal acquisition and processing software is provided, the end user (e.g. medicine doctor) can focus only on the logic part (a give policy) to make it satisfy the needs.

#### FUZZY policies

The real power of the policy-based inference system is that it can easily utilise also some more sophisticated policies and instead of using AGILE policies which on one hand are relatively easy to comprehend even by a person not originating from the computer science domain but on the other hand may be still lacking some flexibility in terms of describing the core decision-making logic, one can for example use FUZZY policies which allow specifying very powerful but flexible at the same time FUZZY rules^91^.

**Figure 13:**
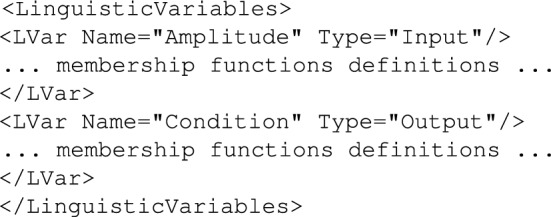
Definition of linguistic variables example.

Using this kind of policies may be a bit more complicated to a person outside of the computer science (control systems theory or even mathematics) domain since FUZZY rules require understanding of fuzzy sets. But FUZZY rules as such add exactly the element which in such a diagnostic/screening system for spine diseases might be crucial: flexibility related to thresholds definition and using terms rather than precise values for linguistic variables while specifying rules for the inference system. par FUZZY policies operate on *FuzzyRules*, *LinguisticVariables* and *MembershipFunctions* which need to be defined by a system-specific expert (since they reflect process specific details, threshold values, etc.). A skeletal containing definition of a *LinguisticVariables* section is shown in Fig. 13.

In the example shown in Fig. 13 there are two linguistic variables defined, an *Input* variable *Error* and an *Output* variable *ControlChange*.

#### *MembershipFunctions* tag

Each linguistic variable may contain a number of definitions for *MembershipFunctions* (denoted as *MF* objects) to specify names and potentially some other parameters of fuzzy sets. For each of the membership functions one must specify its *Name*, *Type* (can be *Trapezoid* or *Gaussian*) and *Value*. Detailed structure for some of these parameters may vary depending on the type of fuzzy system (e.g. Mamdani, Takagi-Sugeno-Kanga, etc.)^92^. For instance, *Trapezoid*-type membership function, the *Value* parameter contains four parameters in order to define parameters of the fuzzy set while in case of *Gaussian*–type membership function the *Value* specifies $c_{i}$ (center) and $\sigma _{i}$ (width) of the fuzzy set $A^{i}$ described by the formula (1):

1 $$\begin{aligned} \mu _{A^i}(x)=\textrm{e}^{-\frac{(c_i-x)^2}{2\sigma _{i}^{2}}} \end{aligned}$$

**Figure 14:**
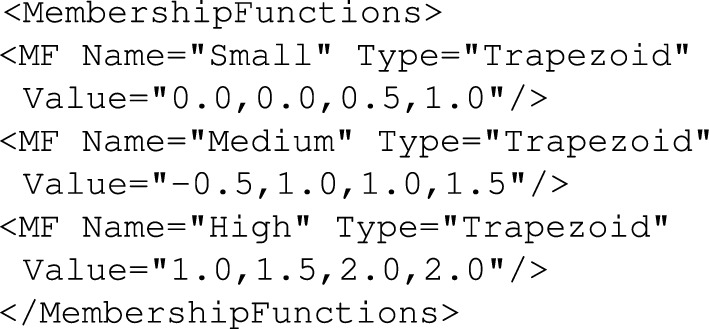
Definition of membership functions example.

Example skeletal definition of *MembershipFunctions* section is presented in Fig. 14.

The above example shows definition of three membership functions (*Small*, *Medium* and *High*) of *Trapezoid* type together with their values.

#### *FuzzyRules* tag

**Figure 15:**
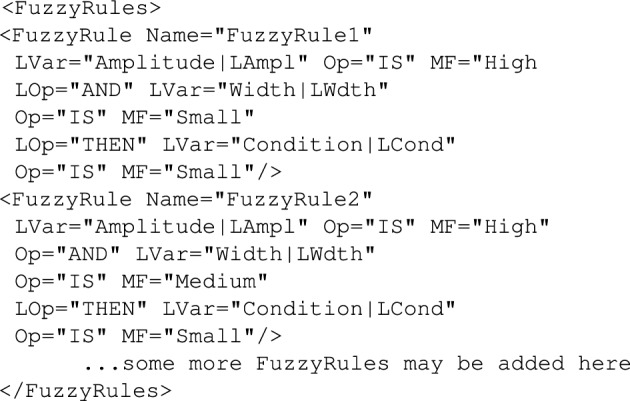
Definition of membership functions example.

Having all the fuzzy reference sets defined, the next step requires building a set of fuzzy rules that would specify details of the fuzzy reasoning. For this purpose the *FuzzyRules* should be used. Within this tag one can define a number *FuzzyRule* objects expressing the details of fuzzy reasoning. Each *FuzzyRule* is of the structure shown in Fig. 15.

Each fuzzy rule defines a mapping between input and output linguistic variables. *LVar* tag is used within a fuzzy rule to specify ties between context variables (representing a physical system quantity) with input linguistic variables for the fuzzification purposes or with output linguistic variables for defuzzification purposes. For example, in the Fig. 14*FuzzyRule1* the two context variables *Amplitude* and *Width* represent the amplitude and width of the recorded signal. These variables are tied with respectively the *LAmpl* and*LWdth* linguistic variables for fuzzification purposes. On the other hand, the *Condition* variable represents severity of the disease and is obtained in the result of defuzzification and it is tied with an aggregated fuzzy output linguistic variable *LCond*.

### Policy-based reasoning

Understanding of what happens in case a healthy individual performs an operation compared to the situation when the same operation is performed by a patient with a spine disease is pretty much all that is needed to put up a reasoning policy.

#### Evaluation of inference system based on AGILE policies

**Figure 16:**
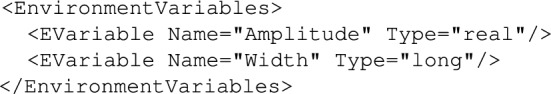
Actual definition of the *EnvironmentVariables*.

To do so what is actually needed is to write this understanding in a form of XML code. First of all, the decision (according to the explanation provided in the “Discussion” Section) is based on the analysis of the amplitude and the duration (width) of the impulse. This automatically implies 2 *EnvironmentVariables*, respectively *Amplitude* and *Width*. Their definition will look as shown in Fig. 16.

**Figure 17:**
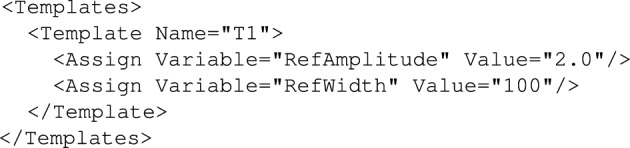
Threshold values initialisation using *Template* object.

Now, having the variables declared the next step would be to define some threshold values for the *EnvironmentVariables* so that the reasoning system would be able to assess whether their actual values are within an allowed range. The most reasonable idea is to use the *InternalVariables* which can be initialised using *Template* object (see Fig. 17).

**Figure 18:**
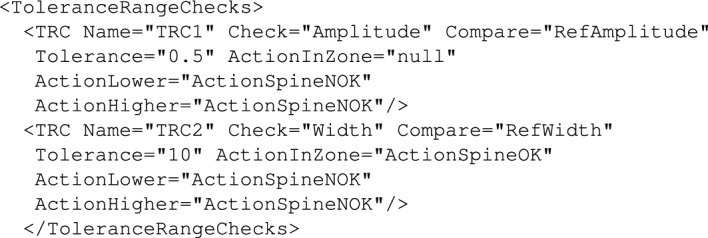
Threshold values initialisation using *Template* object.

The key policy element is always using object(s) which allow various analysis (comparisons to some reference values) of the context information, Depending on the nature of the process either, *Rules*, *ToleranceRangeChecks* or *UtilityFunctions* can be used. In this example we use *ToleranceChecks* which allows defining *dead-zones* for the relevant variables which makes the system make decisions with a certain tolerance to the actual values of state variables. Example declaration of the *ToleranceRangeChecks* which will serve the purpose is provided in Fig. 18.

**Figure 19:**
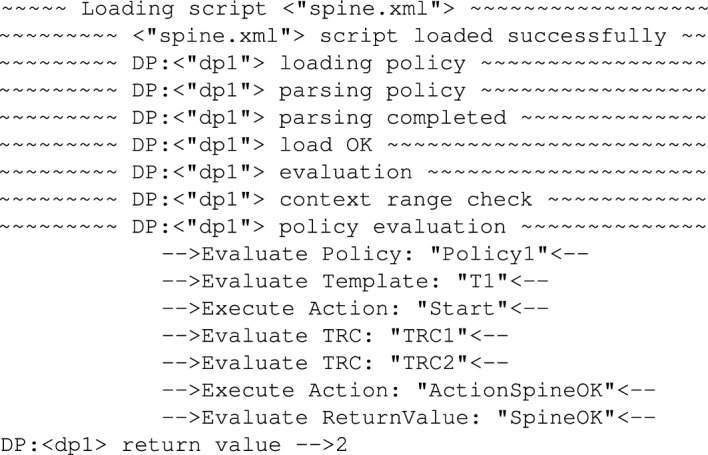
Trace showing policy execution for *Amplitude = 1.6* and *Width = 105*.

Using the provided policy one can test how it responds to the recorded signals. For instance, taking into account the Z-Axis signal from the torso (see Fig. 27) the average maximum amplitude value for the two repeated exercises is around 2 and the average width of the impulse is around 100 samples. So we can set up the reference values for the *Amplitude* and *Width* variables in the policy to reflect that. Testing policy decisions requires running the identified signal parameters (amplitude and width) through the policy using API functions provided. At first the policy response was tested for the provided values of *Amplitude* and *Width* being respectively: 1.6 and 105, which reflects the signal characteristic recorded for the exercise. The results are visible in Fig.19.

As one can see in the Fig. 19, in the policy firstly the *TRC1* was executed which tested if the *Amplitude* value remains within the (+/-) 5 margin from 2. dead-zone. Since the value was within the dead-zone (with *Tolerance = 0.5* indeed the value of 1.6 is not outside the dead-zone), the control was passed to *TRC2* where the pulse width of value 105 was checked. And because this value was within the *Tolerance = 10* then, the policy returned a decision that based on the extracted features provided the spine condition was OK.

**Figure 20:**
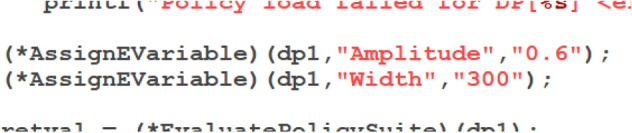
Policy changed to re-define the state variables values.

Second simulation was done with the same policy but different values of *Amplitude* and *Width* passed to the policy, being respectively: 0.6 and 300 samples (which reflects the part of the Fig. 27 recorded for a patient having a spine disease). In the Fig. 20 lines of program code passing the mentioned values to the tested policy are shown.

**Figure 21:**
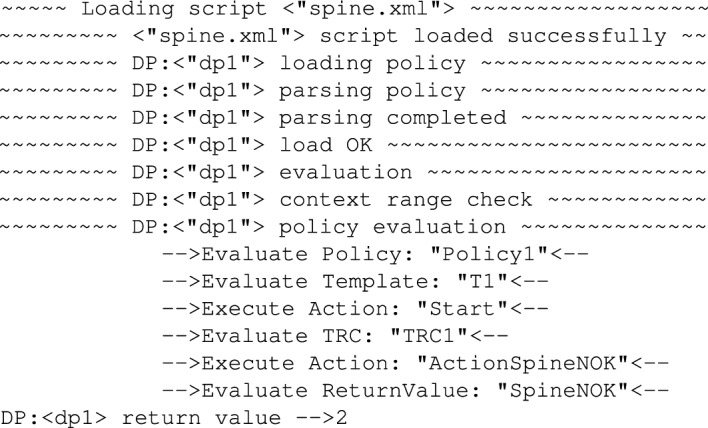
Trace showing policy execution for *Amplitude = 0.6* and *Width = 300*.

The resulting trace showing policy evaluation is shown in the Fig. 21.

As one can see in the Fig. 21, when *TRC1* was evaluated, the actual value of the *Amplitude* was outside of the defined dead-zone, specifically it was lower even taking into account the *Tolerance = 0.5*. In this case immediately the *ActionSpineNOK* was executed and this action returned the decision *SpineNOK*. So the policy tuned to reflect the correct motion pattern indicated by the selected features of the signal was able to recognise the case when the motion pattern deviated from normal.

The provided example was showing analysis of the signal recorded only along one axis and for the IMU placed in the torso area. If the analysis were to be done for all other signals, the policy would be of higher complexity, because it would use more *ToleranceChecks* (and as a result – more *EnvironmentVariables*, *InternalVariables*, *Actions*, etc.). But still, making changes to the way the whole reasoning / inference system works would be still down to editing the policy file using any text editor (as it is a text file) and changing relevant values of variables.

### Experiment design

The experiment conducted for this study purposes was performed in fully simulated conditions where there were actually no patients with different spine issues to be examined (with a specific focus on sagittal imbalance) but instead a healthy person performed a number of physical exercises first, behaving entirely natural and then repeating the exercise with stiffened neck and straighten back. At this stage in our pilot study we focus on evaluation of the hardware and proving that some detectable differences between patients with healthy and unhealthy spine must exist (because in case it does not, it would make no sense to force people who may already feel bad into doing some activity for no reason). Once this stage is finalised, we’ll move toward real tests with real patients.

As it was mentioned before, it is expected that, depending on the spine condition and potentially a specificity of a spine disease, there must be some noticeable/detectable differences first of all, between a motion patterns for a healthy and unhealthy individuals, we designed a simple experiment to collect data in case a person motion pattern is unaffected by any disease and then compare the data with those acquired from a patient who has a certain spine issue (from light ones, like stiff neck or straight back up to some more serious spine conditions, e.g. scoliosis, sagittal imbalance, etc.). To check if this is the case a very simple experiment was designed and measurements were performed in the following way:

- A person was asked to stand up and sit back again from a chair and this motions was supposed to be done in a natural, smooth way;
- After 1 minute the person was asked to repeat the exercise;
- After another minute the person was asked to stand up and sit back again while keeping his head and back as straight and stiff as possible;
- Last time, after another minute has lapsed the person was asked to repeat the stand up and sit back again with his neck and back straight and stiffened;
- 2 IMU units were used, one was placed on the head and another one along the spine in the upper chest area, which means sensors 1 and 3 (see Fig. 3).

The goal was to observe any differences between those two fundamentally different exercises (natural vs. enforced movement which may appear in case there is a spine issue) and, if any, try to assess if there is a pattern characteristic for each of the exercises (hence 2 attempts for each type of exercise) which could potentially be identified and used for diagnostic/screening purposes.

This was not a very complicated experiment yet its goal was to provide data to show the whole diagnostic/screening process, beginning with signal recording through features extraction^93^ (focusing on e.g. selected temporal features and ending with policy-based reasoning.

#### Study participants

For this study purposes – 10 healthy subjects (3 females and 7 males, aged $36 \pm 4.87$; mean height $174 \pm 0.1129$ [m] and mean weight $82.41 \pm 7.43$ [kg].

Informed consent was obtained from all the study participants and the whole experimental procedure was conducted according to the guidelines of the Declaration of Helsinki, approved by the Bioethics Committee of the Nicolaus Copernicus University in Toruń–Collegium Medicum in Bydgoszcz, Poland (protocol code no. KB 416/2008, from 17th September 2008, valid till 31st December 2027).

The exclusion criteria were as follows:

- Medical record regarding any spinal cord diseases;
- Nerve branch block injections in the past;
- Obesity.

### Informed consent

Informed consent was obtained from all the study participants.

## Results

Following the designed experiment, we have recorded a number of signals. Although the IMU unit we used provides a variety of signals, we focused solely on the gyroscope and accelerometer readings. This is due to the fact that they provide different kinds of information. While the gyroscope allows monitoring current body (and hence spine) position/orientation, the accelerometer allows measuring linear acceleration in a given direction (X, Y and Z axes). Both signals allow not only to determine the final body/spine position but also how it was reached (e.g. when a patient performs sit-ups, we can easily determine how the body was moving between the up and down positions).

**Figure 22:**
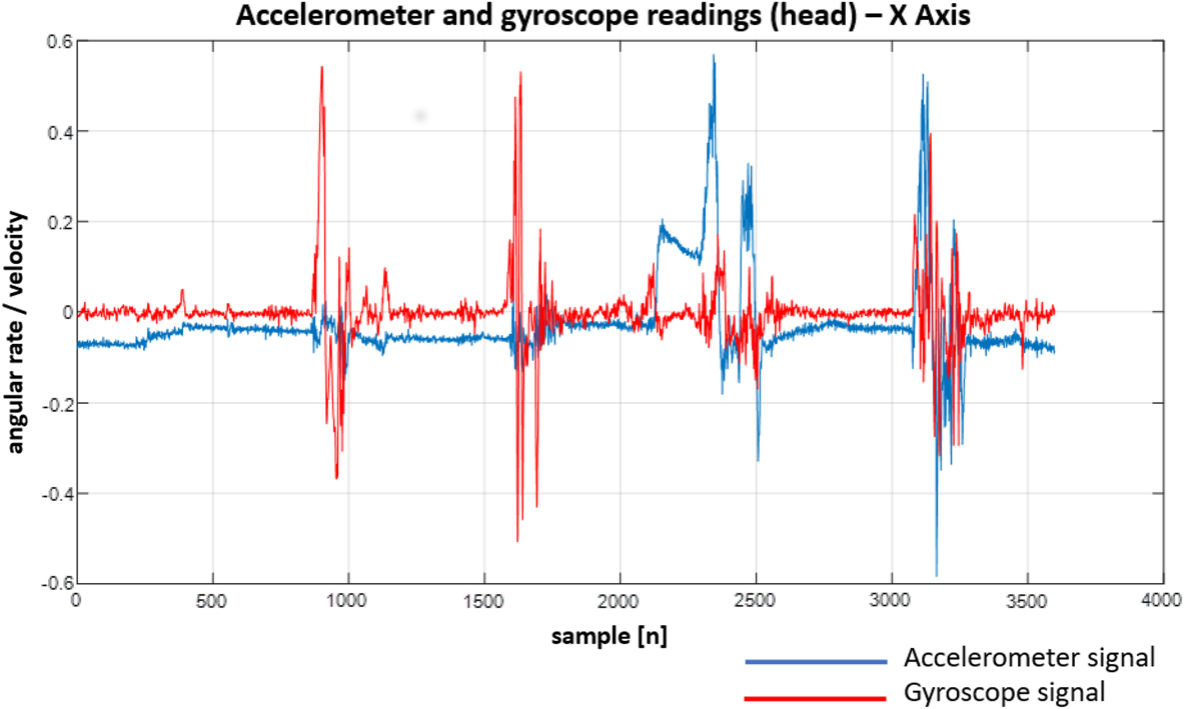
Recordings from the sensors on the head—X Axis.

**Figure 23:**
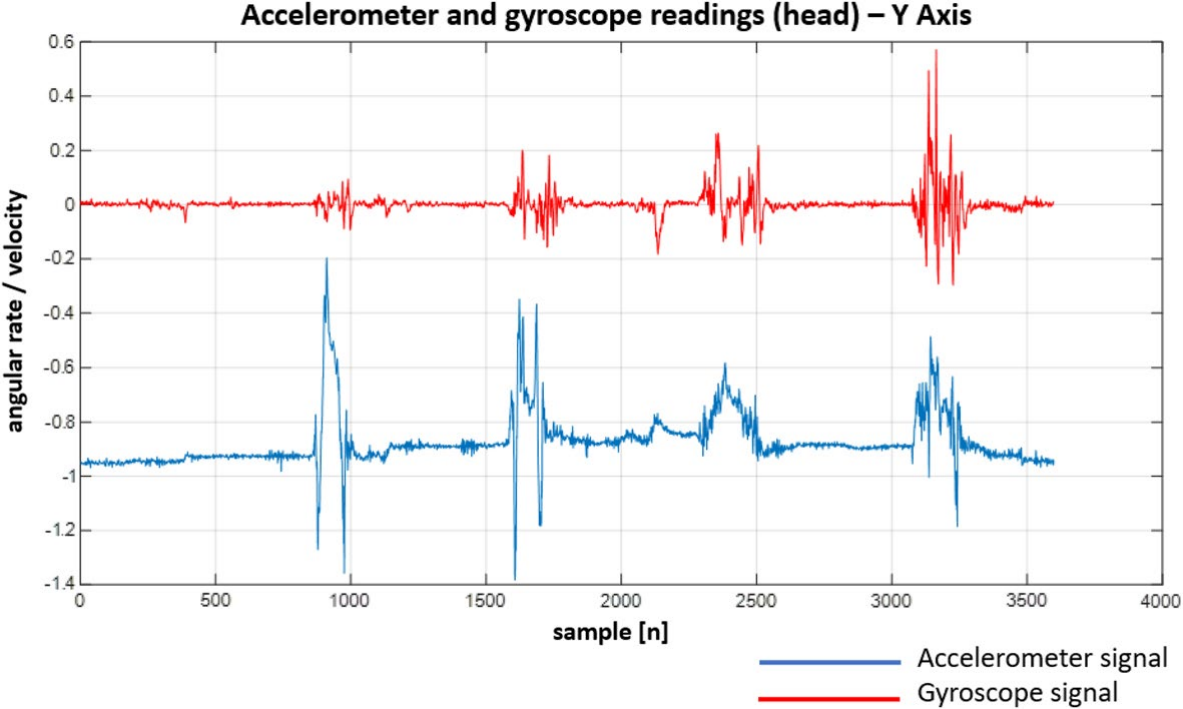
Recordings from the sensors on the head—Y Axis.

**Figure 24:**
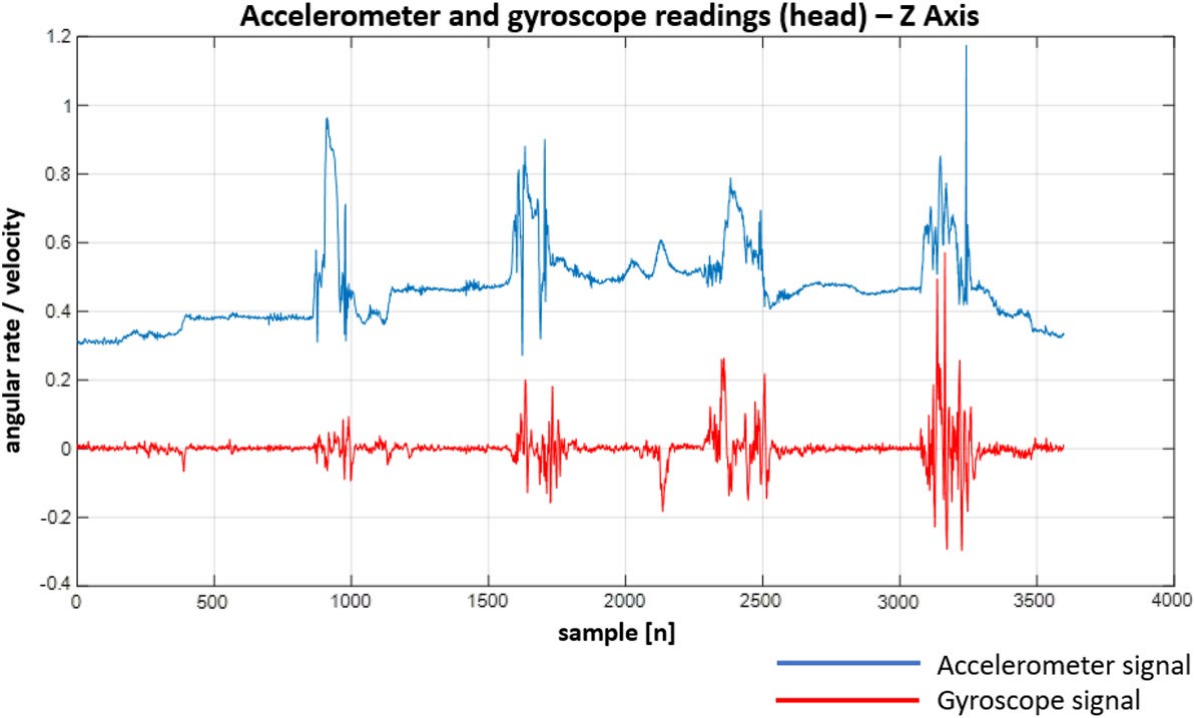
Recordings from the sensors on the head—Z Axis.

**Figure 25:**
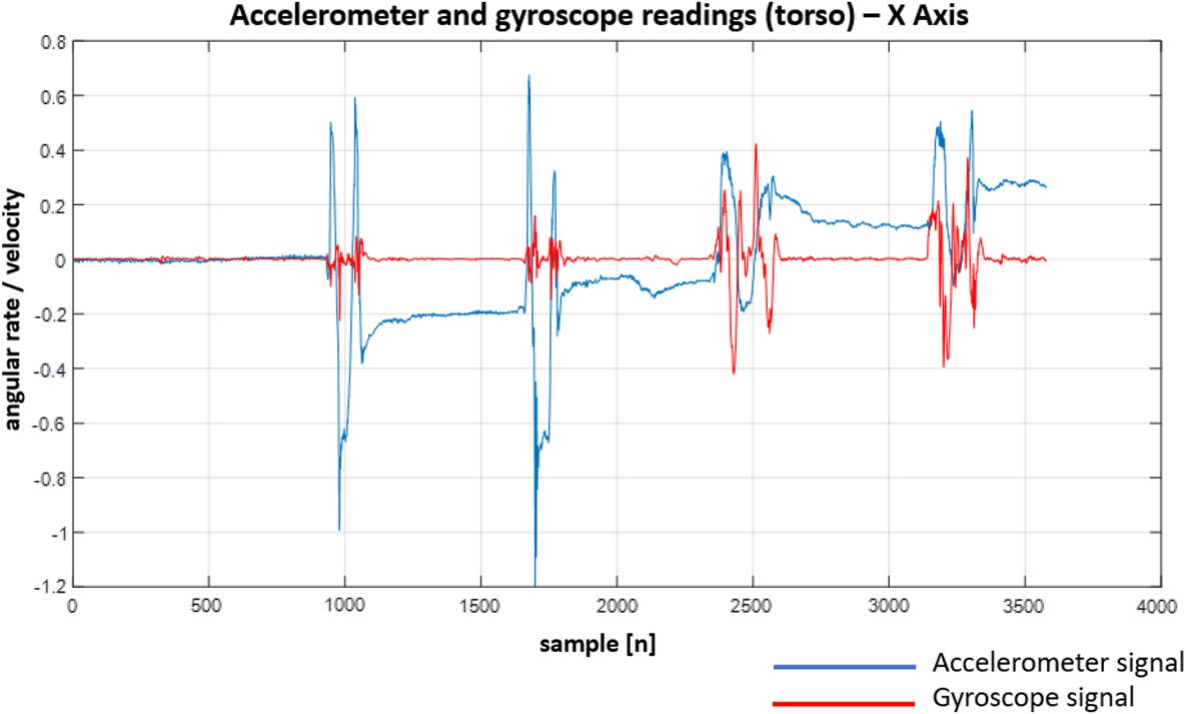
Recordings from the sensors on the torso—X Axis.

**Figure 26:**
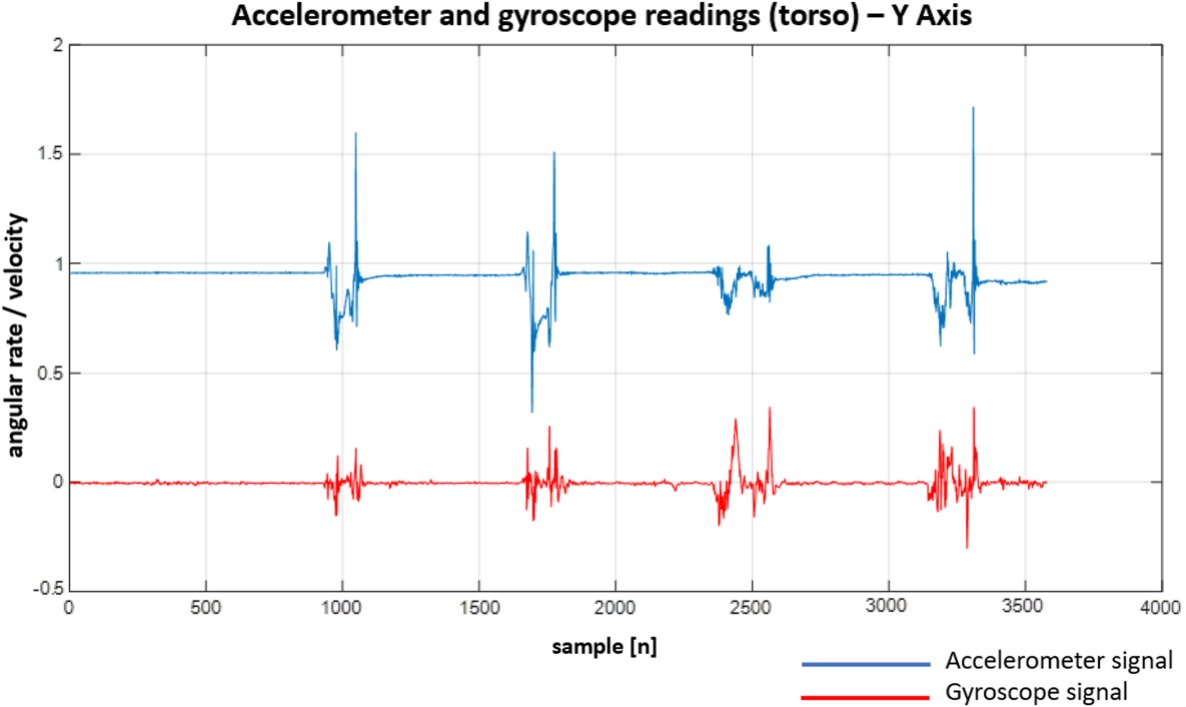
Recordings from the sensors on the torso—Y Axis.

**Figure 27:**
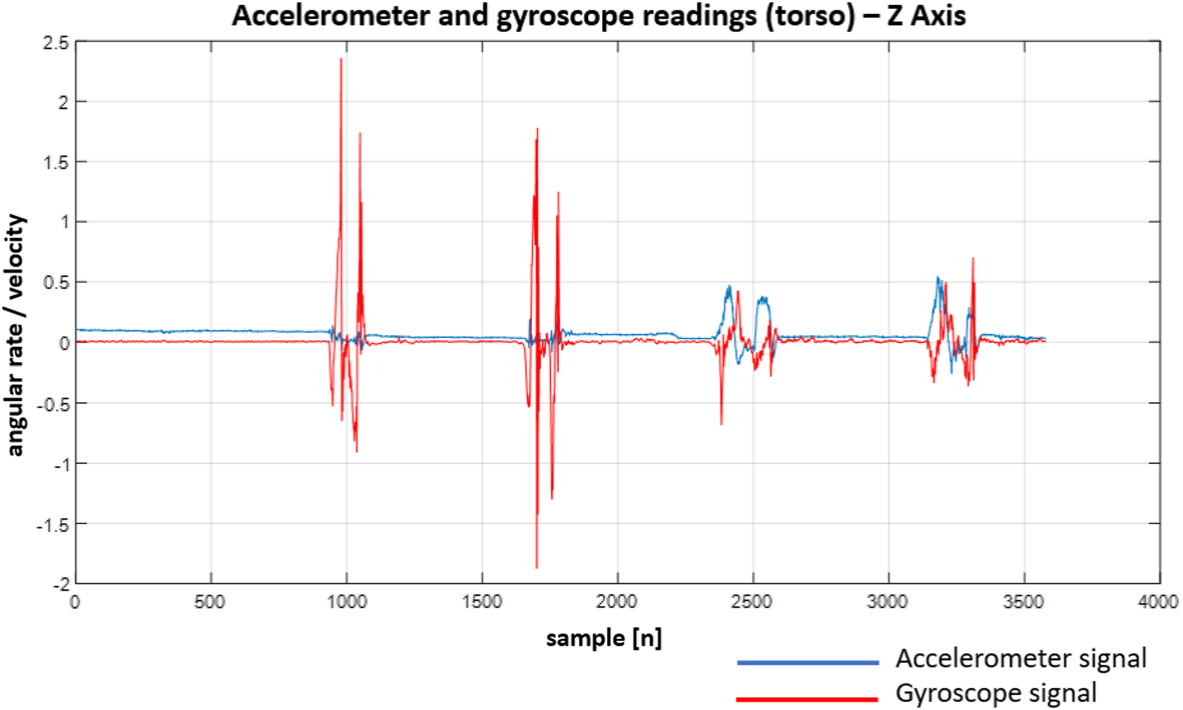
Recordings from the sensors on the torso—Z Axis.

In Figs. 22, 23, 24, 25, 26 and 27 one can see the recorded signals.

## Discussion

When looking at the Figs. 22, 23, 24, 25, 26 and 27 the most interesting readings can be seen whenever the Z–axis from sensors 1 and 3 for the accelerometer and gyroscope is shown (both, in the head and torso area, (see Fig. 3), there is a visible and easy to explain difference between the phase when the patient was doing the exercise normally and in an enforced (indicating a spine disease) manner. So, it is clearly visible from Figs. 24 and 27, that normally the patient does the exercise so that it generates higher amplitude and narrower impulse. This means that having no issues (e.g. indicated by pain) the patient does not make any additional effort to protect his spine (and e.g. to not to increase pain), does the exercise confidently and quickly. While looking at the phase when the exercise reflects enforced (in a specific way which would happen just by itself if the patient would suffer some real spine issues) movement, which is the third and fourth pulse, the amplitude is lower but the whole exercise takes longer to get accomplished. This can be explained as the situation when the patient is careful about his movements, avoids any rapid movements and lets it take longer but without increasing symptoms (e.g. pain).

Much more in-depth knowledge about what exactly happens during the exercise can be derived from analysing signals from the remaining axes and especially – axis X in the head area. What is seen there is that in case there is a normal movement, the patient does not do anything special when doing the exercise, because the values toward the X axes are relatively small. The interesting thing happens in the enforced position: the signal amplitudes become much higher than in the normal movement scenario which may indicate that the patient was not actually doing the stand up and sit down exercise along the Z axis (meaning straight up and then straight down) but instead he seemed to go a little bit sideways. From the force(s) point of view (and forces are vector quantities) explanation would be that if the movement goes only along the Z axis this means that the spine is subject to maximum force vector along the Z axis whereas adding the movement along X axis the resulting force vector affecting the spine will be of lower value hence no/smaller increase in symptoms.

Obviously, the above explanations are purely based on the analysis of some temporal features of the signals and this approach is especially beneficial in relation to all systems performing on-line (real-time) analysis because no complex processing is needed to come up with a diagnosis (or recommendation). On the contrary, systems based e.g. on ML/ANNs require much more time (they are much more computationally heavy) and besides, there is no easy and quick way to adjust the inference system because it requires starting over the whole learning process. Our approach would only require another version to be put together offline and then replacing the currently processed policy with its newer version.

It is not excluded that as one of the future improvements of our method we may potentially adjust our inference system so that it would allow incorporating expert knowledge from different domains (e.g. not only rheumatologist but also physiotherapists, osteopaths, etc.).

### Evaluation of inference system based on FUZZY policies

Alternatively, instead of using AGILE policy in the described scenario, one can also use FUZZY policy. Obviously, defining fuzzy sets (membership functions, their number, ranges, etc.) is usually beyond an expert in medicine, but in case these parameters are set by a person who is familiar with fuzzy logic, then the medicine expert has even easier task to do in case there was a need to adjust the inference system because now the whole adjustment is down to adding/removing/changing a set of *If (...) and (...) THEN (...)* rules. An example FUZZY policy which is addressing the same problem as the previously analysed AGILE policy. In order to illustrate how the diagnostic system works under control of the FUZZY policies we tested it for the same values of *Amplitude* and pulse *Width* as it was in case of AGILE policies.

Rule base for the fuzzy system is shown Table 1.

**Table 1:** Rule Base.

Amplitude			
Width	High	Medium	Small
Small	Small	Small	Medium
Medium	Small	Medium	High
High	Medium	High	High

**Figure 28:**
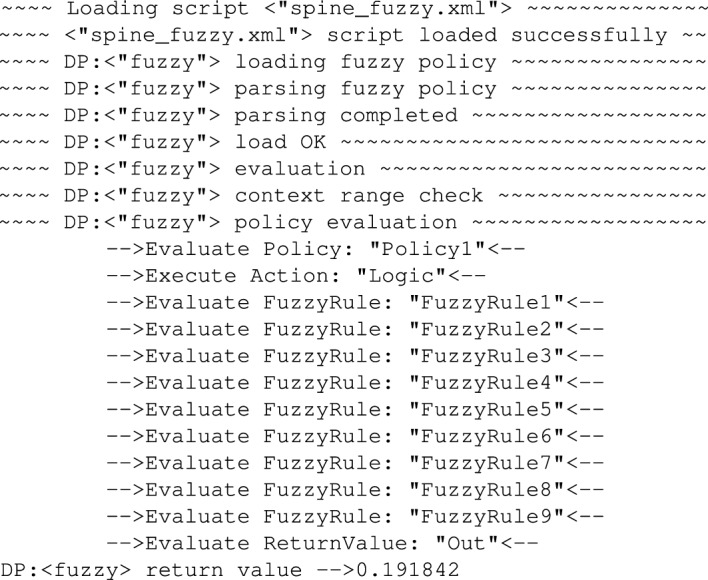
Trace showing policy execution for *Amplitude = 1.6* and *Width = 105*.

So, following the testing procedure presented for AGILE policies we first tested the policy for *Amplitude = 1.6* and pulse *Width = 105*. As we remember, this reflects the situation when the signal was recorded for a healthy individual. AGILE policy in this situation returned the decision that the spine condition for this person is *OK*. The output of the FUZZY policy for the same values is presented in Fig. 28.

Knowing that the FUZZY policy was designed so that the spine disease (condition) severity ranges from 0 (meaning no spine issues whatsoever were detected) to 1 (meaning severe spine condition was detected), the value of *0.191842* is relatively low and indicates that for the extracted features values (*Amplitude* and pulse *Width*) the system does not detect a serious spine condition.

**Figure 29:**
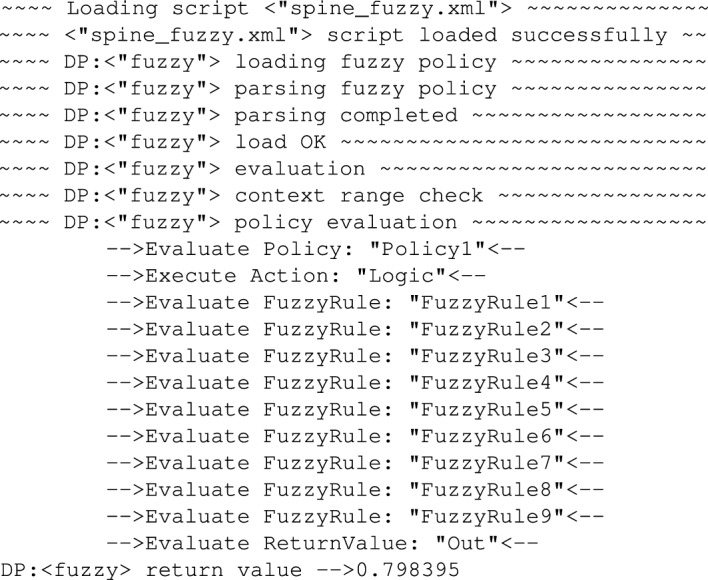
Trace showing policy execution for *Amplitude = 1.6* and *Width = 105*.

In the second test the FUZZY policy was tested again with the same values as the AGILE policy. So, firstly the *Amplitude* and pulse *Width* were set respectively to: 0.6 and 300. The results of the FUZZY policy evaluation are visible in Fig. 29.

As it is presented in the Fig. 28, after evaluating the set of 9 fuzzy rules the system returned decision that for the *Amplitude = 0.6* and pulse *Width = 300* the severity of the disease is at the level of *0.798395*. Taking into account that the scale is 0..1 we can say that it is relatively high. For comparison, in this case the AGILE policy returned the decision *SpineNOK*.

### Study limitations

As our study is of preliminary character and aimed to demonstrate only the idea of using 2-IMU-based systems as an alternative to more sophisticated and invasive diagnostic methods, we decided to focus on showing its potential. Based on a thorough literature study regarding the use of e.g. electromyography (EMG) for appropriate movement pattern assessment^94–97^ and also based on a very wide expertise in spine disease diagnostics and treatment – we assumed that in most cases subjects with back pain were especially challenging to distinguish from the healthy reference group.

Another limitation of the study can be a problem with differentiation of healthy signals from those affected with a disease, which has been presented by Dindorf et al. in their work^23^, where the authors showed that in some cases it is hard to distinguish healthy patterns from those affected with a disease.

In this paper we did not simulate anything. The two different types of movements to which we refer as “healthy” and “unhealthy” ones are only provided to demonstrate that our method is able to distinguish different movement patterns out of which one can be attributed to a “healty”and the other to an “unhealthy” movement type. In reality, one would need to gather signals from a representative sample of healthy and unhealthy subjects to operate on proper representation of the two types of movements.

The limitations of the research are also due to the small research sample, but also to the impact of the following: the COVID-19 pandemic and the war in Ukraine, which translated into the disruption of parts of the supply chains, the availability of electronic parts and software, and the international cooperation in which this project is carried out.

### Directions for further research

Directions for further research include both hardware and software development of the device towards further improvement of performance as part of the development of the Clinic 4.0 paradigm^98^. This includes not only verification through clinical trials on bigger samples, but also obtaining certification under the Medical Devices Regulations (MDR) and ISO 13485^99^.

An important element of our approach is the cyber security of the proposed solution^100,101^. The rapid development of artificial intelligence, including machine learning, is suggesting to us more and more new methods and techniques falling within the framework of computational intelligence that can accelerate and increase the accuracy of analyze, inference and prediction from data^102–104^.

Further research should focus on recommendations compared to the gold standard, for example MRI combined with clinical examination to determine its accuracy.

## Conclusion

A spinal cord disturbances can lead to deterioration of professional work, decreased efficiency in daily life and is associated with sensory and motor defects, respiratory and urinary complications, pressure ulcers, spasms, and pain^105^. The cognitive impairment can develop according to spinal cord failure^106^. The cognitive problems anxiety, depression, and other psychiatric and psychological problems can develop secondary to spinal cord disturbances^107^. Transcutaneous spinal cord can induce cortical and subcortical effects^108^. The direct current spinal stimulation improves locomotor learning in healthy humans^109^. According to the scientific data computations important in cortico-subcortical loop taking place also in the brainstem and spinal cord structure^110^. The data show that the spinal cord plays an important role in mental functions^111^. The study in primates has found that ventral striatum (eminent structure of cortico-subcortical loop) plays a crucial role in the recovery of skilled movement after spinal cord injury, by driving neural activity in primary motor cortex^112^. The spinal cord function may have greater importance to mental functioning than previously thought – which may have important implications for more general neurophysiological and philosophical considerations and support the concept of the embodied mind^112^ and predictive mind^113^.

In this paper we have described an expert system which can be used for screening tests for various spine diseases (e.g. scoliosis, sagittal imbalance, etc.). This system is based on a policy evaluation engine where the policy is a higher level logic which can be used for diagnostic/screening purposes. The key benefit of using this approach is that usually the diagnostic process must take into account various symptoms and depending on their severity/presence/absence/etc. a specific diagnosis can be set. This diagnostic process is usually performed by a medicine doctor. What makes policy-based computing an ideal technology which can be used for spine (or other) diseases diagnostic is that every single policy represents very much the doctor’s (expert) knowledge which is expressed through a number of policy objects and defined operations (e.g. comparisons) performed on these objects (e.g. policy may compare a specific feature extracted from IMU recording with a threshold value and make a decision depending on how one relates to another). In a sense it is a typical *if-then* or *if-then-else* reasoning so much reflecting the typical reasoning process performed by a human being.

Another benefit resulting from our approach is that the policy itself is down to a text file where all the diagnostic/expert knowledge is stored. Such a file can easily be put up or changed (e.g. through a provided policy editing tool) so that ultimately the expert (meaning medicine doctor or a person/medical personnel involved into a diagnostic process) could focus on defining dependencies between symptoms and diseases rather than on some details related to the policy structure itself. Such an approach means that the policy definition (expressing the expert knowledge) and the program logic itself where the policy file will be then used are entirely decoupled. So the software developer can focus on the program side whilst the person performing the diagnostic process can focus on the diagnostic logic only. Having this all working together it is then possible to process much more data in an automated way without or with limited expert supervision. Which is exactly what is needed while doing some screening diagnostic tests where there is usually a lot of data which ideally needs to be processed in a relatively short period of time.

We can safely say that based on the provided example where we recorded signals from head and torso area, along spine, the system is able to provide diagnosis based on the extracted features. Obviously, because the diagnosis comes from an automated diagnostic process, the cases where especially the system detects some issues should be anyway reviewed by a human expert. But as a screening/diagnostic tool it satisfies the requirements.

## Data Availability

Data available upon written request from the corresponding author.
